# Physical Activity, Weight Loss, and Weight Maintenance in the DiOGenes Multicenter Trial

**DOI:** 10.3389/fnut.2021.683369

**Published:** 2021-06-30

**Authors:** Marleen A. van Baak, Gabby Hul, Arne Astrup, Wim H. Saris

**Affiliations:** ^1^Department of Human Biology, NUTRIM School of Nutrition and Translational Research in Metabolism, Faculty of Health, Medicine and Life Sciences, Maastricht University Medical Centre+, Maastricht, Netherlands; ^2^Department of Nutrition, Exercise and Sports, University of Copenhagen, Copenhagen, Denmark

**Keywords:** obesity, exercise, weight regain, diet, metabolic health

## Abstract

In this secondary analysis of the DiOGenes study, we investigated whether physical activity (PA) contributes to diet-induced weight loss and helps to reduce subsequent regain. We also studied the associations of PA with changes in cardiometabolic variables. Adults with overweight were included and followed an 8-week low-calorie diet (LCD). When successful (>8% weight loss), participants were randomized to different *ad libitum* diet groups and were advised to maintain their weight loss over the 6-month intervention period. Body weight (BW), body composition, cardiometabolic variables and subjectively-assessed PA were measured at baseline, at the end of weight loss and at the end of the intervention. BW was reduced by the LCD (from 99.8 ± 16.7 to 88.4 ± 14.9 kg; *P* < 0.001). This reduction was maintained during the weight maintenance period (89.2 ± 16.0 kg). Total PA (sum score of the three subscales of the Baecke questionnaire) increased during the weight loss period (from 8.16 ± 0.83 to 8.39 ± 0.78; *P* < 0.001) and this increase was subsequently maintained (8.42 ± 0.90). We found no evidence that baseline PA predicted weight loss. However, a higher level of baseline PA predicted a larger weight-loss-induced improvement in total cholesterol, triglycerides, glucose and CRP, and in post-prandial insulin sensitivity (Matsuda index). Subsequent weight and fat mass maintenance were predicted by the post-weight loss level of PA and associated with changes in PA during the weight maintenance phase. In conclusion, despite the fact that higher baseline levels of PA did not predict more weight loss during the LCD, nor that an increase in PA during the LCD was associated with more weight loss, higher PA levels were associated with more improvements in several cardiometabolic variables. The positive effect of higher PA on weight loss maintenance seems in contrast to randomized controlled trials that have not been able to confirm a positive effect of exercise training programmes on weight loss maintenance. This analysis supports the notion that higher self-imposed levels of PA may improve the cardiometabolic risk profile during weight loss and help to maintain weight loss afterwards.

## Introduction

The beneficial effects of physical activity (PA) are sufficiently known and include improvements in physical fitness and well-being. In individuals with obesity PA levels are usually low, contributing to low levels of cardiorespiratory fitness and ill metabolic health. Many people with overweight or obesity try to lose weight by means of an energy-restricted diet. In well-structured weight-loss progammes many of them will be successful in losing a substantial amount of weight. However, in the long run maintaining this weight loss turns out to be very difficult (1–4). There is ample evidence from randomized clinical trials (RCTs) that adding an exercise program to a weight loss programme by means of energy restriction will result in extra weight loss [eg., (5–7)]. Combined diet and exercise interventions on the long-term have been found to result in more weight loss than diet alone (8, 9). In contrast, there is no evidence from RCTs that exercise training programmes help to prevent weight regain after weight loss (7, 10). On the other hand, a systematic review of weight control registries shows that an increased level of PA is the most consistent positive correlate of weight loss maintenance (11). Whether self-selected, habitual levels of PA contribute to more weight loss success and may help to prevent weight regain is less well studied.

We therefore performed a secondary analysis of the large multicenter European DiOGenes (Diet, Obesity and Genes) trial (12). Primary aim of this trial was to investigate the effect of different macronutrient compositions of an *ad libitum* diet on weight regain after successful weight loss. In this trial, adults with overweight or obesity were included.

The primary objective of this analysis was to investigate whether self-selected habitual levels of PA contribute to weight loss and weight loss maintenance success. Based on previous literature (5–7, 13), we hypothesized that higher levels of PA are associated with more weight loss during an energy-restricted diet and also with less subsequent weight regain. We therefore analyzed whether baseline PA predicts weight loss during an energy-restricted diet and whether the PA level after weight loss predicts weight maintenance. In addition we analyzed whether PA levels change over time in this weight loss/weight maintenance study, whether weight loss is associated with a change in PA during the weight loss phase, and whether weight maintenance is associated with a change in PA during the weight maintenance period. Where relevant these questions were also addressed for body composition, blood pressure and selected metabolic variables.

## Materials and Methods

### Study Design

DiOGenes is a large multicenter European DiOGenes (Diet, Obesity and Genes) trial, which was conducted between 2006 and 2009 in eight academic centers across Europe (Denmark, Netherlands, Unites Kingdom, Germany, Czech Republic, Greece, Spain, Bulgaria). The trial was registered under ClinicalTrials.gov number NCT00390637. Overweight or obese adults and their children were recruited. After baseline measurements, the adults tried to lose weight by means of a low-calorie diet (~800–1000 kcal/d) for 8 weeks. Participants having successfully lost at least 8% of their initial weight were randomized into one of five groups with different diet compositions: healthy diet, high protein/high glycemic index (GI), high protein/low GI, low protein/high GI, or low protein/low GI. Dietary intake during this period was *ad libitum*. Further details can be found in Larsen et al. (12). The primary aim of the study was to analyze the effect of diet composition on weight maintenance. These primary outcomes have been reported previously (14).

### Subjects

In total 1,121 individuals [men(M) and women(F) with at least one child <18 years in their household] were included in the study. They were generally healthy. Details on in- and exclusion criteria can be found in (12). All participants gave informed consent prior to the study and the study was conducted according to the Declaration of Helsinki. 773 participants that completed the 8-week weight loss phase and had lost ≥8% of their initial body weight were randomized to the subsequent 6-month randomized *ad libitum* diet intervention, which was completed by 548 participants (14). However, not all data were available at all time points, especially the data on PA were missing in many participants. Therefore, the number of subjects will differ in the different analyses. Complete body weight and PA data were available for 193 participants (76M/117F).

### Methods

Measurements where obtained at baseline, at the end of the 8-week weight loss period and at the end of the 6-month weight maintenance period. All measurements were performed in the morning after an overnight fast. Body weight was measured on calibrated digital scales (Seca 861, Hamburg, Germany) to the nearest 0.1 kg. Body composition was measured by dual-energy radiograph absorption (Lunar Radiation, Madison, WI) or bio-impedance (Quad-Scan 4000; Bodystat, Douglas, Isle of Man, United Kingdom) depending on study center. Blood pressure was measured in sitting position after 5 min of rest with a semi-automatic device (Omron). A fasting blood sample was drawn after a 10-min rest from the antecubital vein into vacutainers containing clot activator and gel for serum separation. After 10–30 min coagulation at room temperature, samples were centrifuged at 2500 x g for 15 min at room temperature. Within 30 min of centrifugation, serum was transferred to cryo vials and stored at −80°C until analysis. An oral glucose tolerance test was performed subsequently from which the Matsuda index for postprandial insulin sensitivity was calculated. All blood samples were analyzed in the same lab. Serum total cholesterol, HDL cholesterol, and TG concentrations were quantified by enzyme immunoassays (Ortho-Clinical Diagnostics, Johnson & Johnson) for the Vitros 5.1 FS analyzer. LDL and VLDL cholesterol concentrations were calculated using Friedewald's equation (15). Serum glucose was measured by a colorimetric assay (Ortho-Clinical Diagnostics) for the Vitros 950 analyzer and serum insulin was measured by an immunoassay (Siemens Healthcare Diagnostics) for the ADVIA Centaur XP. Serum CRP was quantified by a high sensitivity immunoassay (hsCRP, Ortho-Clinical Diagnostics) for the Vitros 5.1 FS analyzer with a detection limit of 0.1 mg/L. CRP values >10 mg/L were taken as indications of acute inflammation and the CRP values were excluded from the dataset. Homeostasis model of assessment-insulin resistance (HOMA-IR) was calculated as [fasting glucose (mmol/L) x fasting insulin (mIU/L)]/22.5. The Baecke questionnaire on PA (16) was filled in online at the same time points. Participants were asked to report their PA over the last month. The Baecke questionnaire distinguishes between PA during work, leisure time and sports. For each activity category a mean score was calculated. By adding up the three scores total PA was calculated. The Baecke questionnaire has been validated against energy expenditure measured by objective methods, such as the doubly labeled water technique and tri-axal accelerometry (17, 18).

### Data Analysis

Data are presented as mean ± SD. Changes in PA and anthropometric and metabolic variables over time were analyzed by means of repeated measurements ANOVA. Simple correlations were tested according to Pearson. Regression analysis was used to study predictors of weight and body composition changes with or without adjustment for potential confounders. To analyze whether baseline PA predicted the changes in anthropometric and metabolic variables over the weight loss period, the value of the outcome variable (anthropometric or metabolic variables) at the end of the weight loss period was the dependent variable. As independent variables the baseline values of the outcome variable and total physical activities score were included (model 1). In model 2 we adjusted model 1 for the weight change during the weight loss phase to see whether the association was independent of weight loss. In model 3 we additionally adjusted for sex. A similar approach was used to analyze whether PA at the end of the weight loss phase predicted the changes during the weight maintenance period. SPSS version 25 was used for the statistical analysis.

## Results

### Changes in PA, Body Weight, Body Composition, Blood Pressure, and Metabolic Variables Over Time

As to be expected, ANOVA analyses showed that body weight (BW), body fat percentage (%BF) and fat mass (FM) were significantly reduced by the energy-restricted diet (BW from 99.8 ± 16.7 to 88.4 ± 14.9 kg; %BF from 39.8 ± 8.4 to 35.4 ± 14.9 %; FM from 39.8 ± 11.6 to 31.4 ± 10.5 kg; all *P* < 0.001) and the reductions were maintained during the weight maintenance period (Table 1). Fat free mass (FFM) was also reduced during the weight loss period (from 60.0 ± 12.8 to 57.0 ± 11.7 kg; *P* < 0.001), but it recovered partially during the weight maintenance phase (to 58.1 ± 12.1 kg; *P* < 0.05 vs baseline). Total PA, expressed as the sum score of the three sub scales of the Baecke questionnaire (work, leisure time, and sport) increased during the weight loss period (from 8.16 ± 0.83 to 8.39 ± 0.78; *P* < 0.001) and this increase was maintained during the weight maintenance period (Table 1). The increase in total activity was mainly due to an increase in leisure time activity. Work and sport activity did not change significantly (Table 1).

**Table 1:** Anthropometrics and scores for physical activity categories and total physical activity at the three measurement time points (*N* = 193).

**Variable**	**Baseline** **mean**	**Baseline** **SD**	**End of weight loss mean**	**End of weight loss SD**	**End of weight maintenance mean**	**End of weight maintenance SD**	***P* value**^**a**^
BW (kg)	99.8	16.7	88.4^b^	14.9	89.2^b^	16.0	0.000
BF% (%)	39.8	8.4	35.4^b^	8.9	34.7^b^	8.7	0.000
FM (kg)	39.8	11.6	31.4^b^	10.5	31.2^b^	10.5	0.000
FFM (kg)	60.0	12.8	57.0^b^	11.7	58.1^b^^,^^c^	12.1	0.000
Work score	2.74	0.34	2.76	0.34	2.76	0.37	0.355
Leisure time score	2.78	0.65	3.02^b^	0.63	3.04^b^	0.67	0.000
Sport score	2.64	0.37	2.60	0.40	2.61	0.37	0.306
Total score	8.16	0.83	8.39^b^	0.78	8.42^b^	0.90	0.000

a *P value from repeated measurements ANOVA*;

b *significantly different from baseline (post-hoc paired t-test with Bonferroni correction, P < 0.001)*;

c *significantly different from end of weight loss (post-hoc paired t-test with Bonferroni correction, P < 0.001). BW, body weight; BF%, percent body fat; FM, fat mass; FFM, fat free mass*.

Blood pressure and metabolic variables were all significantly improved at the end of the weight loss phase (Table 2). Blood pressure, total, LDL-, and HDL-cholesterol had returned to baseline levels after the weight maintenance phase. Triglycerides, glucose, insulin, and HOMA-IR also increased during the weight maintenance phase, but remained lower than baseline. The Matsuda index and CRP levels maintained the weight-loss-induced levels (Table 2). Data on males and females separately can be found in the Supplementary Material.

**Table 2:** Blood pressure and metabolic parameters at the three measurement time points (*N* = 107).

**Variable**	**Baseline** **mean**	**Baseline** **SD**	**End of weight loss mean**	**End of weight loss SD**	**End of weight maintenance mean**	**End of weight maintenance SD**	***P* value**^**a**^
SBP (mm Hg)	125	14	116^b^	14	123^c^	14	0.000
DBP (mm Hg)	77	11	72^b^	10	76^c^	10	0.000
total cholesterol (mmol/L)	4.7	1.0	4.0^b^	0.9	4.8^c^	0.9	0.000
LDL-cholesterol (mmol/L)	2.9	0.9	2.5^b^	0.7	3.0^c^	0.9	0.000
HDL-cholesterol (mmol/L)	1.2	0.3	1.1^b^	0.2	1.3^c^	0.3	0.000
Triglycerides (mmol/L)	1.3	0.6	1.0^b^	0.4	1.2^b^^,^^c^	0.5	0.000
Glucose (mmol/L)	5.1	0.5	4.8^b^	0.4	4.9^b^^,^^c^	0.4	0.000
Insulin (μIU/L)	11.7	7.1	6.7^b^	6.1	7.8^b^^,^^c^	6.5	0.000
HOMA_IR	3.1	1.9	1.7^b^	1.7	2.0^b^^,^^c^	1.5	0.000
Matsuda index	4.9	2.5	8.5^b^	4.0	8.2^b^	4.0	0.000
CRP (mg/L)	3.3	2.5	2.3^b^	2.4	1.9^b^	2.4	0.000

a *P value from repeated measurements ANOVA*;

b *significantly different from baseline (post-hoc paired t-test with Bonferroni correction, P < 0.01)*;

c *significantly different from end of weight loss (post-hoc paired t-test with Bonferroni correction, P < 0.05). SBP, systolic blood pressure; DBP, diastolic blood pressure; HOMA-IR, HOMA index for insulin resistance; CRP, C-reactive protein*.

### Association Between Baseline PA and Changes During the Weight Loss Phase

**Figure 1:**
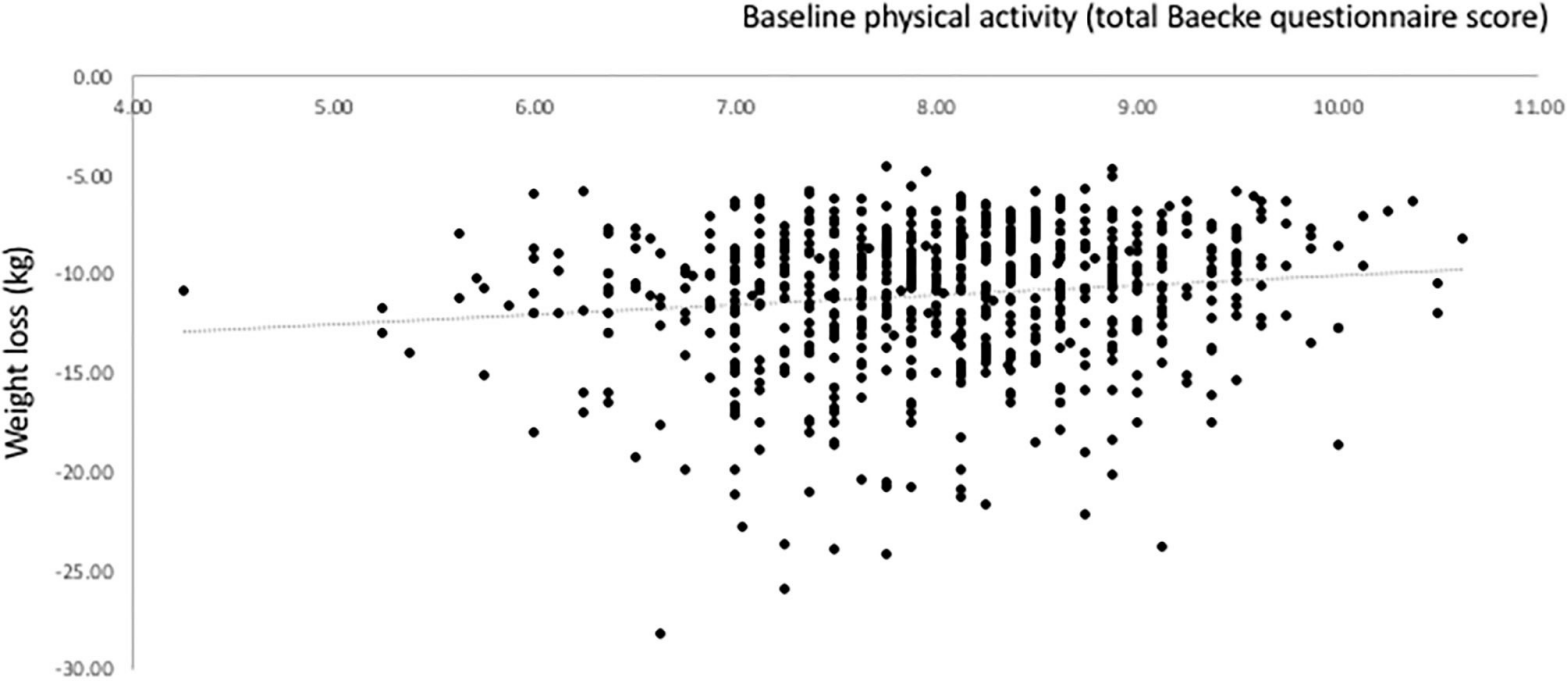
Relationship between baseline physical activity (PA) and weight loss induced by an energy-restricted diet in participants of the DiOGenes trial.

The association of baseline PA with weight loss during the weight loss phase is shown in Figure 1. The association is positive (*r* = 0.132, *P* = 0.000), suggesting that those with higher baseline PA lost less weight. However, multiple regression analysis showed no statistically significant evidence for an association between baseline PA and weight at the end of the weight loss phase, when baseline weight was included as a covariate (Table 3). Furthermore, regression analyses for the outcome variables FM, SBP, DBP, insulin, HOMA-IR and HDL cholesterol showed no significant influence of baseline PA. However, baseline PA significantly predicted the change in CRP, total cholesterol, triglycerides, glucose, and the Matsuda index. The change in FFM and LDL-cholesterol also tended to be predicted by baseline PA (*P* < 0.10). A higher PA at baseline was associated with larger improvements in these variables, also when adjusted for weight loss and sex (Table 3).

**Table 3:** Results of the regression analyses with total physical activity at baseline as the independent variable and different variables at the end of the weight loss phase as the dependent variable.

	Model 1^*^				Model 2^**^			Model 3^***^		
**Dependent**	***r***^**2**^	**B**	**SE**	***P***	**B**	**SE**	***P***	**B**	**SE**	***P***
**Variable**										
BW (kg)	0.969	0.008	0.116	0.946				0.021	0.114	0.856
BF%	0.767	0.015	0.190	0.939	−0.097	0.188	0.608	−0.220	0.186	0.238
FM (kg)	0.883	0.099	0.204	0.628	−0.013	0.188	0.944	−0.048	0.188	0.799
FFM (kg)	0.887	−0.270	0.189	0.155	−0.318	0.189	0.093	−0.332	0.186	0.075
SBP (mm Hg)	0.435	0.052	0.430	0.903	−0.087	0.431	0.839	−0.087	0.431	0.840
DBP (mm Hg)	0.490	0.392	0.299	0.190	0.291	0.300	0.332	0.292	0.298	0.329
total cholesterol (mmol/L)	0.495	−0.050	0.027	0.070	−0.065	0.027	0.018	−0.065	0.027	0.017
LDL-cholesterol (mmol/L)	0.535	−0.031	0.023	0.174	−0.042	0.023	0.066	−0.043	0.023	0.065
HDL-cholesterol (mmol/L)	0.553	0.006	0.008	0.405	0.005	0.008	0.493	0.006	0.008	0.422
Triglycerides (mmol/L)	0.289	−0.053	0.016	0.001	−0.058	0.016	0.000	−0.059	0.016	0.000
Glucose (mmol/L)	0.310	−0.078	0.018	0.000	−0.082	0.018	0.000	−0.081	0.018	0.000
Insulin (μIU/L)	0.666	−0.075	0.216	0.731	−0.178	0.217	0.411	−0.182	0.216	0.401
HOMA-IR	0.585	−0.050	0.068	0.464	−0.076	0.069	0.268	−0.077	0.069	0.261
Matsuda index	0.365	0.449	0.134	0.001	0.510	0.134	0.000	0.513	0.133	0.000
CRP (mg/L)	0.509	−0.200	0.091	0.028	−0.198	0.092	0.031	−0.195	0.092	0.034

* *model 1 adjusted for the baseline value of the variable*;

** *model 2 additional adjustment for the weight loss*;

*** *model 3 additional adjustment for sex. r^2^, coefficient of determination of model 1; B, regression coefficient; SE, standard error; P, P value. BW, body weight; BF%, percent body fat; FM, fat mass; FFM, fat free mass; SBP, systolic blood pressure; DBP, diastolic blood pressure; HOMA-IR, HOMA index for insulin resistance; CRP, C-reactive protein*.

We also analyzed whether a change in PA during the weight loss phase of the study was associated with weight loss. No correlation was found for body weight (*r* = −0.052, *P* = 0.306, *N* = 395), nor for the change in FM or the change in FFM.

### The Level of PA After Weight Loss and Weight Maintenance Success

**Figure 2:**
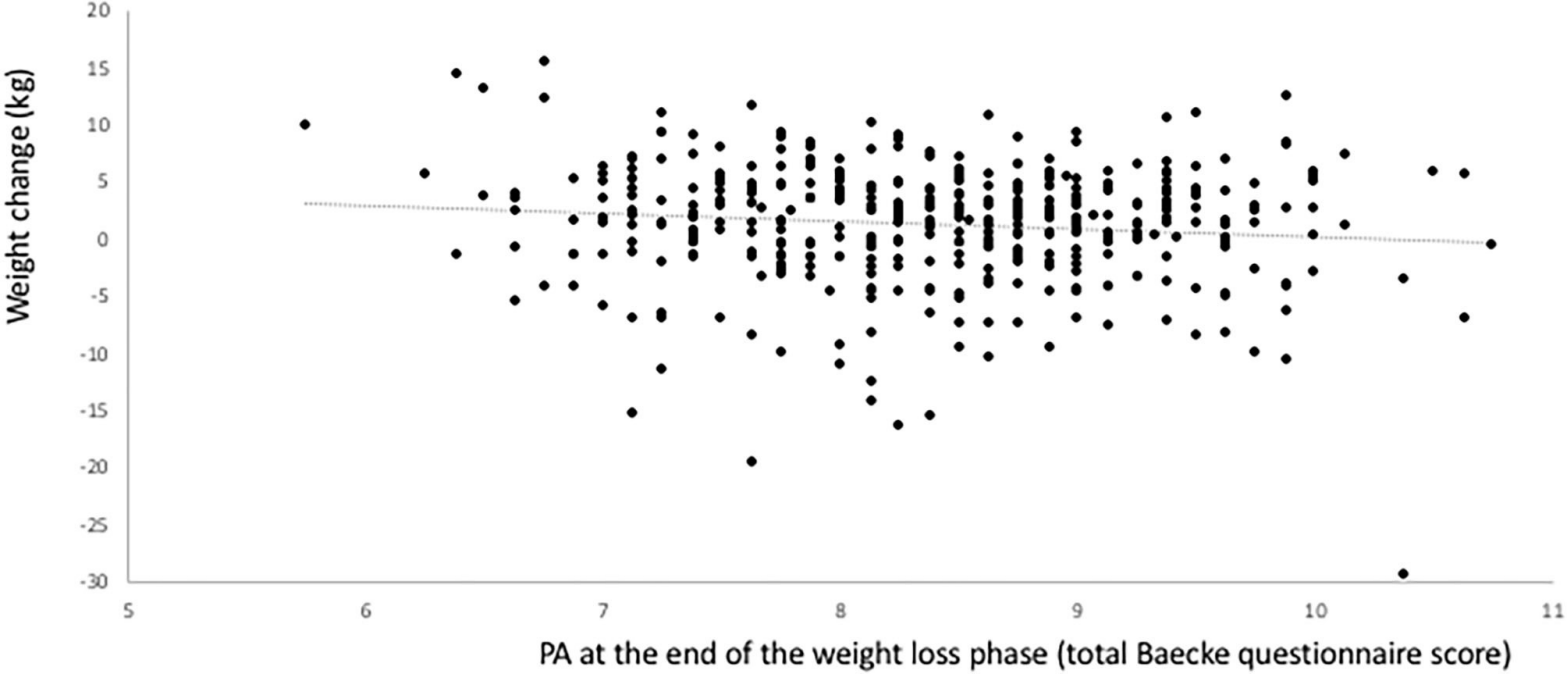
Relationship between the level of physical activity at the end of the weight loss phase (start of the weight maintenance period) and subsequent weight changes during the weight maintenance phase in the DiOGenes trial.

The relationship between the level of PA at the end of the weight loss phase and the subsequent weight changes are shown in Figure 2. The association is negative, indicating that the higher the PA level at the end of weight loss, the smaller the weight regain or the larger the further weight loss. In a multiple linear regression analysis, PA at the end of the weight loss phase significantly predicted body weight at the end of the 6-month intervention period adjusted for body weight at the end of weight loss (B −0.675, SE 0.277, *P* = 0.015, *N* = 421) (Table 4). There was no interaction with the diet group to which the participant was randomized. Negative associations were present for FM and FFM as well, although not statistically significant (both *P* > 0.05). No significant associations with CRP, glucose, HOMA-IR, insulin, LDL, Matsuda, HDL, TG, SBP, and DBP were detected and there was no interaction with diet in any of the associations.

**Table 4:** Results of the regression analyses with total physical activity at the end of the weight loss phase as the independent variable and the variable at the end of the weight maintenance phase as the dependent variable.

	Model 1^*^				Model 2^**^			Model 3^***^		
**Dependent variable**	***r*^**2**^**	**B**	**SE**	***P***	**B**	**SE**	***P***	**B**	**SE**	***P***
BW (kg)	0.890	−0.677	0.284	0.017				−0.613	0.276	0.027
BF%	0.810	−0.187	0.236	0.429	0.100	0.193	0.605	−0.104	0.190	0.582
FM (kg)	0.801	−0.384	0.297	0.196	0.129	0.182	0.477	0.045	0.182	0.806
FFM (kg)	0.929	−0.337	0.198	0.090	−0.192	0.182	0.291	−0.066	0.174	0.705
SBP (mm Hg)	0.381	−0.378	0.595	0.526	−0.049	0.581	0.932	0.342	0.571	0.550
DBP (mm Hg)	0.484	−0.079	0.427	0.854	0.129	0.419	0.759	0.284	0.415	0.494
total cholesterol (mmol/L)	0.352	−0.026	0.042	0.537	0.004	0.042	0.918	0.015	0.042	0.717
LDL- cholesterol (mmol/L)	0.422	0.000	0.037	0.998	0.027	0.037	0.469	0.038	0.037	0.304
HDL-cholesterol (mmol/L)	0.493	0.004	0.013	0.738	0.001	0.013	0.945	−0.007	0.013	0.600
Triglycerides (mmol/L)	0.298	−0.050	0.025	0.043	−0.031	0.024	0.199	−0.016	0.024	0.511
Glucose (mmol/L)	0.298	0.004	0.023	0.858	0.015	0.023	0.513	0.019	0.023	0.401
Insulin (μIU/L)	0.688	−0.501	0.331	0.131	−0.401	0.332	0.228	−0.330	0.332	0.321
HOMA-IR	0.661	−0.127	0.088	0.151	−0.100	0.089	0.262	−0.078	0.088	0.375
Matsuda index	0.345	0.218	0.186	0.242	0.116	0.179	0.515	0.070	0.179	0.695
CRP (mg/L)	0.252	0.045	0.111	0.686	0.094	0.110	0.394	0.030	0.109	0.787

* *model 1 adjusted for the baseline value of the variable*;

** *model 2 additional adjustment for the weight regain*;

*** *model 3 additional adjustment for diet group and sex. r^2^, coefficient of determination of model 1; B, regression coefficient; SE, standard error; P, P value. BW, body weight; BF%, percent body fat; FM, fat mass; FFM, fat free mass; SBP, systolic blood pressure; DBP, diastolic blood pressure; HOMA-IR, HOMA index for insulin resistance; CRP, C-reactive protein*.

We also analyzed whether the change in PA over the weight maintenance period was associated with changes in weight or body composition. A significant negative association was found for body weight and FM, but not for FFM.

## Discussion

In this secondary analysis of the DiOGenes trial, we found that the level of PA at baseline did not predict weight loss induced by an energy-restricted diet. However, weight-loss induced improvements in CRP, total cholesterol, triglycerides, glucose and the Matsuda index were more pronounced in participants with higher baseline PA, independent of the change in body weight. On the other hand, the PA level reported at the end of the weight loss phase significantly predicted weight and FM maintenance, but not FFM maintenance or the change in any of the other measured variables. No interaction with diet composition during the weight maintenance phase was found.

Despite the fact that the DiOGenes trial did not include an intervention on PA levels of the participants, the results show an increase in self-reported total PA during the weight-loss phase, which was maintained during the weight maintenance phase. The increase was mainly due to an increase in leisure time PA as measured by the Baecke questionnaire, whereas the sport index did not change. The leisure-time PA index of the Baecke questionnaire is based on the frequency of television viewing, walking, and cycling and on the time spent walking or cycling for transportation. The sports index is derived from the two most frequently played sports with an estimation of their intensity and the number of hours per week and of months per year these two sports are played by the subject, an estimation of the level of PA compared with that of the subject's age peers, the frequency of sweating during leisure time, and the frequency of playing sports. The scores on the leisure time index and sport index were not correlated at baseline (*r* = −0.005, *P* = 0.883, *N* = 917), suggesting that they clearly measure different aspects of PA.

Although, in contrast to the full DiOGenes population (*n* = 548) (14), there was no statistically significant average weight regain in participants with complete data for PA, weight and body composition presented in Table 1, whereas the interindividual variation was large as shown in Figure 2.

Two systematic reviews, based on RCTs, addressed the question whether adding an exercise intervention to an energy-restricted diet intervention would improve total weight loss individuals with obesity (19, 20). In the meta-analysis by Cheng et al. which focused on postmenopausal women with obesity, the diet plus exercise intervention groups reduced their body weight by −1.2 kg more than the diet-only groups. Weight loss was on average approximately 6.5 kg in the diet-only group, with study durations varying between 12 weeks and 1 year (19). In the meta-analysis of Sardeli et al., which included elderly individuals with obesity, resistance training did not result in extra weight loss, but prevented the FFM loss induced by the energy-restricted diet. Average weight loss was around 4 kg with study durations varying between 12 and 24 weeks (20). Apart from the specific populations that were addressed in these reviews, it is obvious that both total weight loss and rate of weight loss were considerably higher in the DiOGenes study than in the studies included in these two systematic reviews. It is possible that the larger energy restriction and larger weight loss in the DiOGenes study explains why there was no association between the PA level at baseline or the spontaneous increase in PA during the weight loss intervention and body weight loss: the effect of the energy restriction may have been pre-dominant and the role of PA too subtle to induce measurable effects. It is therefore intriguing that we did find that participants who were more active at baseline did show more improvements in CRP, total cholesterol, triglycerides, glucose and the Matsuda index. This may suggest that a more active lifestyle makes the body more sensitive to weight-loss induced changes in these parameters.

In contrast to the lack of effect of baseline PA on weight loss, we found a negative association between the level of PA at the end of the 8-week weight loss phase and the weight and FM regain over the subsequent 6 months. None of the other variables was associated with the level of PA at this time point. During the 6-month weight maintenance period participants were asked to try to at least maintain their body weight loss and not regain weight. In addition, the effect of different diet compositions, which varied in macronutrient composition, was tested. The association between PA and weight regain was independent of diet composition. A somewhat similar analysis was performed previously in the STORM trial (13). This trial investigated whether continued treatment with sibutramine had an effect on weight regain (18-months follow-up) after a 6-month period of weight loss induced by a combination of an energy-restricted diet and sibutramine treatment. PA was also measured by the Baecke questionnaire. A secondary analysis showed that a higher average PA level over the follow-up period was associated with better weight maintenance, expressed as weight regain/weight loss (13). This was the same in the DiOGenes trial (*r* = 0.124, *P* = 0.041). On the other hand, a systematic review and meta-analysis of randomized clinical trials published in 2014 that investigated the effect of exercise interventions on weight loss maintenance concluded that there was no effect of different forms of exercise training on weight regain after weight loss based on 4 RCT's (10). Two of the RCT's applied aerobic training and the other two resistance training. A more recent systematic review (7) was able to include one additional RCT applying a 1-year resistance training in post-menopausal women after weight loss (21), which also showed no effect on weight, FM, and FFM regain. Nevertheless, it cannot be excluded that there is a difference between self-selected levels of PA and those driven by an exercise intervention where intrinsic motivation and adherence may be a problem. This was also hinted at by Foright et al. (22).

As suggested in Foright et al. (22), the beneficial effect of higher levels of PA on weight maintenance may be related to a reduction of the weight-loss-induced gap between energy intake and energy expenditure that promotes weight regain. The mechanisms underlying this effect are not fully clear, but may include effects of exercise on GI signals, increased capacity for *de novo* lipogenesis in the liver and a reduction in adipose tissue, increased insulin response to a glucose load, increased dietary fat oxidation in the muscle and preservation of muscle mass and increases in leptin and insulin sensitivity in the brain (22). A recent systematic review and meta-analysis suggests that exercise interventions do not lead to measurable increases in daily energy intake in people with overweight or obesity (23), which would support some of the suggested mechanisms. Another proposed mechanism is a higher FFM associated with a higher level of PA, which leads to a higher level of resting energy expenditure. Indeed, the baseline level of PA was positively correlated with FFM, after adjustment for baseline weight and sex (B 0.505, SE 0.218, *P* = 0.021).

The volume of exercise or PA needed for prevention of weight regain after weight loss is not clear. Current recommendations vary between 200 and 450 min/week (15, 24, 25), but these are mainly based on cross-sectional, non-randomized and retrospective studies. A recently published RCT (26) compared the effects of three different partially supervised exercise programs (150, 220, or 300 min/week) in combination with weekly behavioral counseling on weight regain over 12 months after a weight loss program where participants had lost ≥5% of their initial weight. No difference in weight regain was found among the groups. The lack of a control group does not allow conclusions about whether or how much weight regain was prevented. The authors concluded from these results that it is likely that less exercise is needed than currently recommended for prevention of weight regain (26). From the current study no conclusions can be drawn about the effective volume of self-selected PA, because the questionnaire used to assess PA does not allow quantification of the volume of PA. This is clearly an area that needs further study.

Although this analysis has the advantage that it is based on a considerable number of participants, a major limitation is that PA was derived from a questionnaire. Although the Baecke questionnaire has been validated against total energy expenditure as measured by the doubly labeled water technique and other objective techniques on a group level (17, 18), this does not necessarily mean that the reported values are reliable on an individual level. Moreover, it was not validated specifically in a population with overweight and obesity. Although subgroups of the DiOGenes population participated in measurements of total daily energy expenditure by means of doubly labeled water or the IDEEA device [Intelligent Device for Energy Expenditure and PA (IDEEA)], these measurements turned out to be too incomplete to validate the questionnaire data. Thus, our results should only be regarded as a starting point for further research with more objective measurement of PA. Furthermore, the DiOGenes trial was designed as a randomized clinical trial. However, this secondary analysis is observational and cause-effect conclusions cannot be drawn from the associations reported. Nevertheless, the results give rise to some interesting hypotheses that could be tested in new studies. The first suggestion is to test whether a higher level of baseline PA makes the body more sensitive to weight-loss-associated changes in several metabolic variables, such as plasma glucose, total cholesterol, triglycerides and CRP concentrations and postprandial insulin sensitivity (Matsuda index). The second is to test whether a higher level of self-selected PA after weight loss is associated with better weight loss maintenance than an imposed exercise regimen.

In conclusion, this secondary observational analysis of the DiOGenes RCT found no evidence that baseline PA predicted weight loss induced by a low-calorie diet. However, a higher level of baseline PA predicted a larger weight-loss-induced improvement in plasma levels of total cholesterol, triglycerides, glucose and CRP, and in post-prandial insulin sensitivity (Matsuda index). Subsequent weight and FM maintenance were predicted by the post-weight loss level of PA and associated with changes in PA during the weight maintenance phase. Despite the fact that RCT's so far have not been able to confirm a positive effect of exercise training programmes on weight loss maintenance, this analysis supports the notion that self-imposed levels of PA may help to maintain weight loss.

## Data Availability Statement

Access to the data presented in this article can be requested from: m.vanbaak@maastrichtuniversity.nl.

## Ethics Statement

The studies involving human participants were reviewed and approved by local ethics committees in 8 European countries. The patients/participants provided their written informed consent to participate in this study.

## Author Contributions

MB conceived the idea for the analysis. MB and GH performed the analysis and wrote the manuscript. AA and WS read and commented on the manuscript and conceived the DiOGenes project. WS was the coordinator of the full DiOGenes project and AA the coordinator of research line 1 of the DiOGenes project in which the research on which this manuscript is based was carried out. MB was the principal investigator of the Maastricht center and GH was one of the investigators. All authors contributed to the article and approved the submitted version.

## Conflict of Interest

The authors declare that the research was conducted in the absence of any commercial or financial relationships that could be construed as a potential conflict of interest.
