# Supplementary material for: Physical Activity, Weight Loss, and Weight Maintenance in the DiOGenes Multicenter Trial
Source: Front Nutr. 2021 Jun 30;8:683369. doi: 10.3389/fnut.2021.683369 (PMC8277960; doi:10.3389/fnut.2021.683369)
Supplement: Supplementary file 1 [file Data_Sheet_1.docx]

Supplement Table 1. Anthropometrics and scores for physical activity categories and total physical activity at the three measurement time points for male participants (N = 76).

| **Variable** | **baseline**  **mean** | **baseline**  **SD** | **end of weight loss mean** | **end of weight loss SD** | **end of weight maintenance mean** | **end of weight maintenance SD** | **P value^a^** |
| --- | --- | --- | --- | --- | --- | --- | --- |
| **BW (kg)** | 107.6 | 15.9 | 94.8^b^ | 14.4 | 97.4^b,d^ | 15.6 | 0.000 |
| **BF% (%)** | 32.3 | 7.2 | 27.5^b^ | 5.9 | 27.2^b^ | 5.9 | 0.000 |
| **FM (kg)** | 35.3 | 11.9 | 26.7^b^ | 9.6 | 27.1^b^ | 9.9 | 0.000 |
| **FFM (kg)** | 72.3 | 8.1 | 68.1^b^ | 7.6 | 70.2^c,d^ | 8.1 | 0.004 |
| **work score** | 2.75 | 0.35 | 2.77 | 0.35 | 2.79 | 0.32 | 0.465 |
| **leisure time score** | 2.70 | 0.62 | 2.92^c^ | 0.62 | 2.95^c^ | 0.70 | 0.000 |
| **sport score** | 2.57 | 0.33 | 2.55 | 0.43 | 2.57 | 0.39 | 0.885 |
| **total score** | 8.06 | 0.74 | 8.29^c^ | 0.67 | 8.36^c^ | 0.92 | 0.001 |

^a^ P value from repeated measurements ANOVA; ^b^ significantly different from baseline (post-hoc paired t-test with Bonferroni correction, P < 0.001); ^c^ significantly different from baseline (post-hoc paired t-test with Bonferroni correction, P < 0.05); ^d^ significantly different from end of weight loss (post-hoc paired t-test with Bonferroni correction, P < 0.01). BW, body weight; BF%, percent body fat; FM, fat mass; FFM, fatfree mass.

Supplement Table 2. Anthropometrics and scores for physical activity categories and total physical activity at the three measurement time points for female participants (N = 118).

| **Variable** | **baseline**  **mean** | **baseline**  **SD** | **end of weight loss mean** | **end of weight loss SD** | **end of weight maintenance mean** | **end of weight maintenance SD** | **P value^a^** |
| --- | --- | --- | --- | --- | --- | --- | --- |
| **BW (kg)** | 96.7 | 15.3 | 84.3^b^ | 13.8 | 84.0^b^ | 14.0 | 0.000 |
| **BF% (%)** | 44.6 | 4.9 | 40.5^b^ | 6.3 | 40.5^b^ | 6.3 | 0.000 |
| **FM (kg)** | 42.7 | 10.4 | 34.5^b^ | 9.8 | 33.8^b^ | 10.0 | 0.000 |
| **FFM (kg)** | 52.0 | 6.9 | 49.7^b^ | 7.3 | 50.2^b^ | 6.2 | 0.000 |
| **work score** | 2.74 | 0.34 | 2.77 | 0.33 | 2.75 | 0.35 | 0.616 |
| **leisure time score** | 2.82 | 0.66 | 3.09^c^ | 0.63 | 3.09^b^ | 0.64 | 0.000 |
| **sport score** | 2.66 | 0.40 | 2.61 | 0.40 | 2.61 | 0.39 | 0.301 |
| **total score** | 8.23 | 0.88 | 8.46^c^ | 0.84 | 8.45^c^ | 0.88 | 0.001 |

^a^ P value from repeated measurements ANOVA; ^b^ significantly different from baseline (post-hoc paired t-test with Bonferroni correction, P < 0.001); ^c^ significantly different from baseline (post-hoc paired t-test with Bonferroni correction, P < 0.01). BW, body weight; BF%, percent body fat; FM, fat mass; FFM, fatfree mass.

Supplement Table 3. Blood pressure and metabolic parameters at the three measurement time points in male participants (N = 49).

| **Variable** | **baseline**  **mean** | **baseline**  **SD** | **end of weight loss mean** | **end of weight loss SD** | **end of weight maintenance mean** | **end of weight maintenance SD** | **P value^a^** |
| --- | --- | --- | --- | --- | --- | --- | --- |
| **SBP (mm Hg)** | 131 | 11 | 119^b^ | 12 | 130^c^ | 13 | 0.000 |
| **DBP (mm Hg)** | 81 | 10 | 73^b^ | 9 | 78^c^ | 9 | 0.000 |
| **total cholesterol (mmol/L)** | 4.8 | 1.2 | 4.0^b^ | 1.0 | 4.9^c^ | 1.0 | 0.000 |
| **LDL- cholesterol (mmol/L)** | 3.1 | 1.1 | 2.6^b^ | 0.8 | 3.1^c^ | 1.0 | 0.000 |
| **HDL-cholesterol (mmol/L)** | 1.0 | 0.2 | 1.0 | 0.2 | 1.2^b,c^ | 0.3 | 0.000 |
| **Triglycerides (mmol/L)** | 1.6 | 0.7 | 1.0^b^ | 0.4 | 1.4^c^ | 0.6 | 0.000 |
| **Glucose (mmol/L)** | 5.2 | 0.6 | 4.9^b^ | 0.4 | 5.1^d^ | 0.5 | 0.001 |
| **Insulin (µIU/L)** | 13.3 | 6.7 | 7.0^b^ | 6.8 | 8.2^b^ | 5.1 | 0.000 |
| **HOMA_IR** | 3.6 | 2.0 | 1.8^b^ | 2.2 | 2.2^b^ | 1.4 | 0.000 |
| **Matsuda index** | 4.2 | 2.2 | 8.5^b^ | 4.3 | 7.5^b^ | 4.1 | 0.000 |
| **CRP (mg/L)** | 3.0 | 2.4 | 2.5 | 2.5 | 1.9^b^ | 1.7 | 0.004 |

^a^ P value from repeated measurements ANOVA; ^b^ significantly different from baseline (post-hoc paired t-test with Bonferroni correction, P < 0.01); ^c^ significantly different from end of weight loss (post-hoc paired t-test with Bonferroni correction, P < 0.001); ^d^ significantly different from end of weight loss (post-hoc paired t-test with Bonferroni correction, P < 0.05). SBP, systolic blood pressure; DBP, diastolic blood pressure; HOMA-IR, HOMA index for insulin resistance; CRP, C-reactive protein.

Supplement Table 4. Blood pressure and metabolic parameters at the three measurement time points in female participants (N = 74).

| **Variable** | **baseline**  **mean** | **baseline**  **SD** | **end of weight loss mean** | **end of weight loss SD** | **end of weight maintenance mean** | **end of weight maintenance SD** | **P value^a^** |
| --- | --- | --- | --- | --- | --- | --- | --- |
| **SBP (mm Hg)** | 121 | 14 | 114^b^ | 12 | 118^d^ | 12 | 0.000 |
| **DBP (mm Hg)** | 75 | 11 | 72^b^ | 10 | 73 | 10 | 0.002 |
| **total cholesterol (mmol/L)** | 4.6 | 0.8 | 4.0^b^ | 0.7 | 4.7^c^ | 0.9 | 0.000 |
| **LDL- cholesterol (mmol/L)** | 2.8 | 0.7 | 2.4^b^ | 0.6 | 2.9^c^ | 0.8 | 0.000 |
| **HDL-cholesterol (mmol/L)** | 1.3 | 0.3 | 1.2^b^ | 0.2 | 1.4^b,c^ | 0.3 | 0.000 |
| **Triglycerides (mmol/L)** | 1.1 | 0.5 | 0.9^b^ | 0.3 | 1.0^b^ | 0.4 | 0.000 |
| **Glucose (mmol/L)** | 5.0 | 0.6 | 4.7^b^ | 0.4 | 4.8^b,d^ | 0.4 | 0.000 |
| **Insulin (µIU/L)** | 10.5 | 7.2 | 6.8^b^ | 5.4 | 7.5^b^ | 6.8 | 0.000 |
| **HOMA_IR** | 2.8 | 2.0 | 1.7^b^ | 1.3 | 1.8^b^ | 1.5 | 0.000 |
| **Matsuda index** | 5.5 | 3.1 | 8.1^b^ | 3.6 | 8.6^b^ | 3.9 | 0.000 |
| **CRP (mg/L)** | 3.5 | 2.6 | 2.2^b^ | 2.2 | 1.9^b^ | 1.9 | 0.000 |

^a^ P value from repeated measurements ANOVA; ^b^ significantly different from baseline (post-hoc paired t-test with Bonferroni correction, P < 0.01); ^c^ significantly different from end of weight loss (post-hoc paired t-test with Bonferroni correction, P < 0.001); ^d^ significantly different from end of weight loss (post-hoc paired t-test with Bonferroni correction, P < 0.05). SBP, systolic blood pressure; DBP, diastolic blood pressure; HOMA-IR, HOMA index for insulin resistance; CRP, C-reactive protein.
